# Insights into research on myocardial ischemia/reperfusion injury from 2012 to 2021: a bibliometric analysis

**DOI:** 10.1186/s40001-022-00967-7

**Published:** 2023-01-09

**Authors:** Ming Bai, Jingjing Zhang, De Chen, Mengying Lu, Junfen Li, Zheng Zhang, Xiaowei Niu

**Affiliations:** 1 Heart Center, The First Hospital of Lanzhou University, Lanzhou, Gansu China; 2 Gansu Clinical Medical Research Center for Cardiovascular Diseases, The First Hospital of Lanzhou University, Lanzhou, Gansu China; 3 Gansu Key Laboratory of Cardiovascular Diseases, The First Hospital of Lanzhou University, Lanzhou, Gansu China; 4 Center for Medical Genetics, Gansu Provincial Clinical Research Center for Birth Defects and Rare Diseases, Gansu Provincial Maternity and Child-Care Hospital, Lanzhou, Gansu China; 5 The First School of Clinical Medicine, Lanzhou University, Lanzhou, Gansu China

**Keywords:** Myocardial ischemia/reperfusion injury, Bibliometric analysis, CiteSpace, VOSviewer, Research hotspots

## Abstract

**Background:**

Numerous studies on myocardial ischemia/reperfusion (MI/R) injury have been undertaken in recent years. Hotspots and developmental trends in MI/R research are being rapidly updated. However, there has been no bibliometric analysis that systematically evaluates existing literature on MI/R injury. Our study explores developments in MI/R research over the past decade, and provides a reference for future research.

**Materials and methods:**

Both experimental and clinical publications on MI/R injury from 2012 to 2021 were retrieved from the Web of Science Core Collection database. The CiteSpace and VOSviewer tools were used to perform a bibliometric analysis.

**Results:**

A total of 8419 papers were analyzed. The number of annual publications demonstrated an overall upward trend, rising from 629 publications in 2012 to 1024 publications in 2021. China, the USA, Germany, England, and Italy were the top five contributors to MI/R studies. The Fourth Military Medical University in China contributed the most publications (188, 2.23%), while the University College London in England cooperated the most with relevant research institutions. Derek J Hausenloy (University College London), Derek M Yellon (University College London), and Gerd Heusch (University of Essen Medical School) were the top three most active and influential scholars according to the H-index. Among the top 10 journals with the most publications, *Basic Research in Cardiology* had the highest impact factors. The top three co-cited journals were *Circulation*, *Circulation Research*, and *Cardiovascular Research*. According to a co-cited reference analysis, MI/R research can be divided across 10 major subfields of mitophagy, cardioprotection, inflammation, remote ischemic preconditioning, long non-coding RNA, melatonin, postconditioning, mitochondria, microvascular obstruction, and ferroptosis. After 2018, the keywords with strongest citation bursts included extracellular vesicles, long non-coding RNA, cell proliferation, microRNA, mitochondrial quality control, mitophagy, biomarker, and mitochondrial biogenesis.

**Conclusions:**

The present study reveals the influential authors, cooperating institutions, and main research foci in the field of MI/R injury in the past decade. The latest hotspots are a more in-depth insight into the molecular mechanisms underlying MI/R injury, such as mitochondrial quality control, non-coding RNAs, cell proliferation, and extracellular vesicles.

**Supplementary Information:**

The online version contains supplementary material available at 10.1186/s40001-022-00967-7.

## Introduction

Ischemic heart disease has remained a leading cause of morbidity and mortality across the world [1]. Restoration of blood flow (reperfusion) is a key treatment for salvaging the ischemic myocardial tissue [2–4]. However, the process of reperfusion itself can induce secondary cardiac dysfunction, namely, myocardial ischemia/reperfusion (MI/R) injury, including myocardial stunning [5], no-reflow phenomenon [6, 7], reperfusion arrhythmias, and lethal reperfusion injury [2, 3]. Numerous experimental and clinical studies have confirmed that, to some extent, MI/R injury mitigates the beneficial effects of reperfusion in cases of acute myocardial infarction and cardiac surgery [2, 3]. MI/R injury contributes to the final infarct size after reperfusion, continuously high rates of heart failure, and adverse clinical outcomes in patients with acute myocardial infarction [2, 3]. In the past decade, relevant data have emerged that enable understanding the pathophysiology of MI/R injury, and exploring the role of adjunct cardioprotection therapy in addition to reperfusion, as well as their underlying signal transduction pathways [2, 3]. Currently, there is no effective therapeutic approach to prevent MI/R injury in clinical settings [8–10]. This emphasizes that the understanding of the complexities of MI/R injury is incomplete [8–10]. Therefore, it would be greatly significant to systematically analyze the literature on MI/R injury, and provide a roadmap for developing effective therapeutic strategies for reperfusion injury.

Bibliometrics has been widely employed to understand the knowledge base, and to explore developmental trends and research frontiers in various research fields through qualitative and quantitative analyses of the scientific literature [11]. In addition, the scientific output and impact of different countries, institutions, journals, and scholars can be evaluated through bibliometric analysis [11]. The bibliometric analysis method plays a vital role in providing valuable references or guidance for scientific research [11]. Several bibliometric studies have helped researchers in quickly obtaining scientific information on the development and progression of cardiovascular diseases. In a bibliometric analysis of heart failure [12], Zhang et al. found that epidemiology, treatment, comorbidity, and atrial fibrillation were the main research directions in the past two decades. Moreover, they identified chronic microvascular inflammation as the latest research paradigm. Ma et al., who also used the bibliometric analysis method, showed that research on exosomes in cardiovascular diseases has focused on ischemic heart disease, pathogenesis, regeneration, stem cells, targeted therapy, biomarkers, and cardiac protection, and these would serve as topics of future research [13]. However, there have been few attempts to evaluate MI/R research using the bibliometric analysis method.

The present study used the bibliometric tools of CiteSpace and VOSviewer [14] for the following purposes: (1) identifying the cooperation and impact of various authors, countries, institutions, and journals, (2) displaying the basic knowledge and developmental trends in the MI/R field through a co-cited reference analysis, and (3) detecting research frontiers for the MI/R field through a keyword analysis. This bibliometric analysis shall provide researchers a multifaceted perspective on MI/R research in the past decade, and lay the foundation for in-depth research on MI/R injury.

## Materials and methods

### Search strategy

The Web of Science Core Collection (WoSCC) database includes the leading global academic journals, and allows downloading full citation records with new records being updated every day. The WoSCC is the most commonly used database for bibliometric analyses. Previous reports have widely confirmed the validity of bibliometric analyses based solely on the WoSCC database [12, 13, 15]. Therefore, we chose the WoSCC database to retrieve relevant studies using an advanced search strategy. The primary search words and combinations were “myocardial reperfusion injury” and “myocardial ischemia/reperfusion injury”. The following were the selection criteria: (1) publication year: January 2012–December 2021, (2) document type: article or review, and (3) language: English. All searches were completed and downloaded on the same day (April 3, 2022). Details of the retrieval procedure are provided in Additional file 1: Fig. S1.

### Data collection

The search results were screened independently by two investigators to exclude irrelevant, duplicate, and withdrawn articles. Disagreements were resolved by consulting a third investigator.

### Data analysis

The WoSCC literature analysis report was used to evaluate publication characteristics, such as output in the form of number of annual publications, journals, authors, citation frequency, impact factor (IF), and Hirsch index (H-index). IF reflects a journal's rank or importance by calculating the average number of times that its selected papers are cited in a particular year. The H-index, indicating that the given author/country has published at least H papers which have each been cited at least H times, was used to evaluate the scientific impact of author or country. GraphPad Prism 7.00 (GraphPad Software, San Diego, USA) was used to analyze publication trends. The growth of publications in the following year was estimated using the polynomial model: $f\left(x\right)=a{x}^{3}+b{x}^{2}+cx+d$.

CiteSpace, a visualization tool developed by Professor Chaomei Chen, analyzes emerging trends and critical changes in scientific literature [16]. We used the CiteSpace V 5.8.R3 software (Drexel University, Philadelphia, USA) to explore collaboration networks between countries/institutes/authors, perform co-cited reference analysis, and identify keywords with citation bursts [11]. The betweenness centrality for each node in a network was calculated to reveal how likely it is for an arbitrary shortest path to go through the node. The node with a centrality value > 0.1 was found to connect two or more large groups of nodes by being in between them [11]. The burst detection algorithm was used to find out how an abrupt change takes place in an event within a specific duration. In addition, the co-cited references were clustered using the log-likelihood ratio test to identify the major subfields of MI/R injury research. Two indicators, including the Modularity Q (*Q* value) and Mean Silhouette (*S* value), were used to evaluate the quality of the cluster network formed. If the *Q* > 0.3, the cluster structure obtained was significant; when the *S* > 0.7, the clustering was efficient and convincing [11].

The VOSviewer (Leiden University, Gravenhage, the Netherlands) software creates, visualizes, and explores knowledge maps based on network data. We used VOSviewer 1.6.18 to identify co-cited journals, and display the network structure [11].

## Results

### Temporal trends in publications

**Fig. 1:**
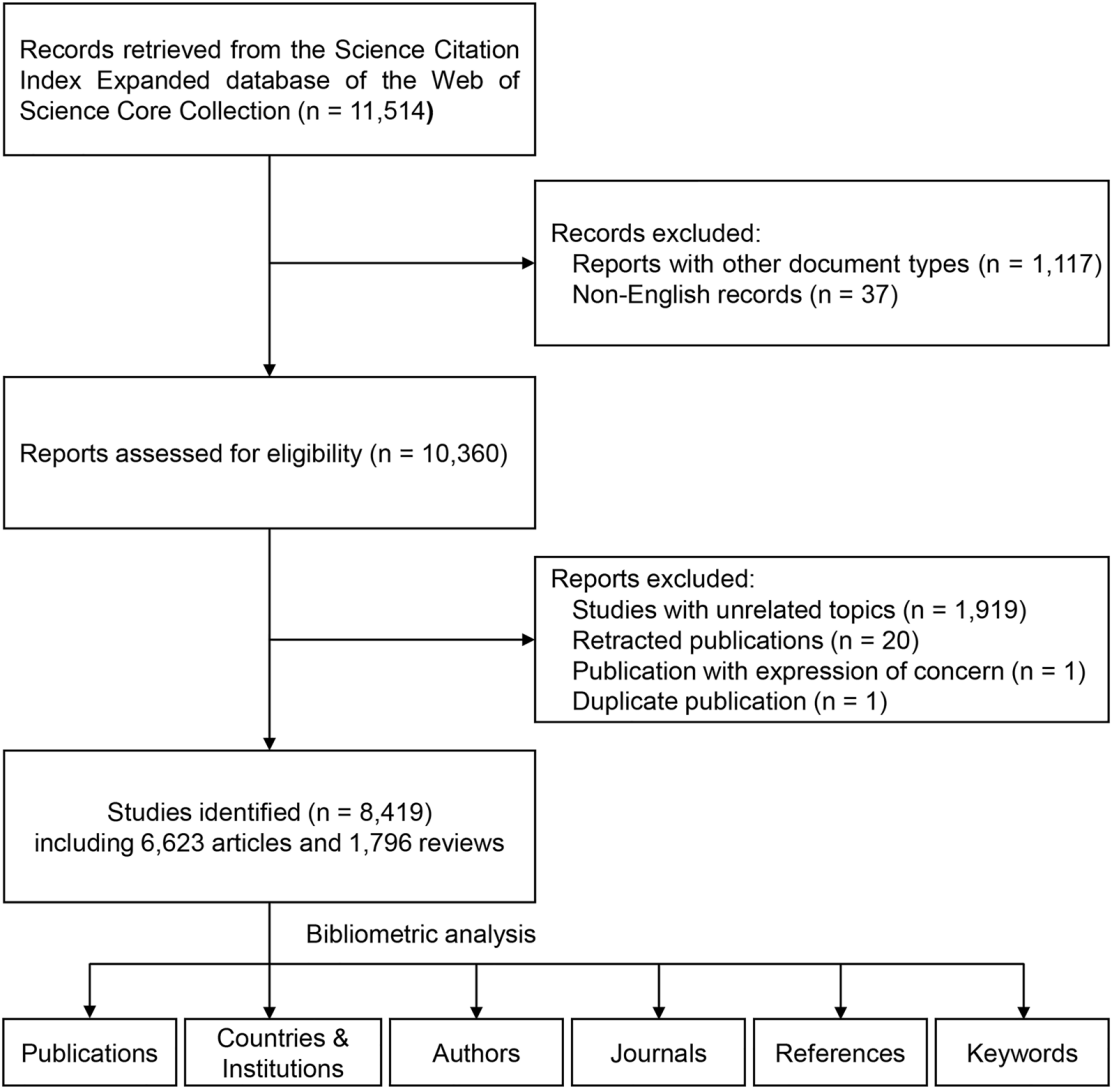
Flowchart of literature selection

After scanning, a total of 8419 English records related to MI/R injury were included, of which 6623 were articles (78.7%) and 1796 were reviews (21.3%). The total citation frequency was 214,942, the H-index count was 156, and the average number of citations per item was 25.53. A flowchart of the literature selection is presented in Fig. 1.

**Fig. 2:**
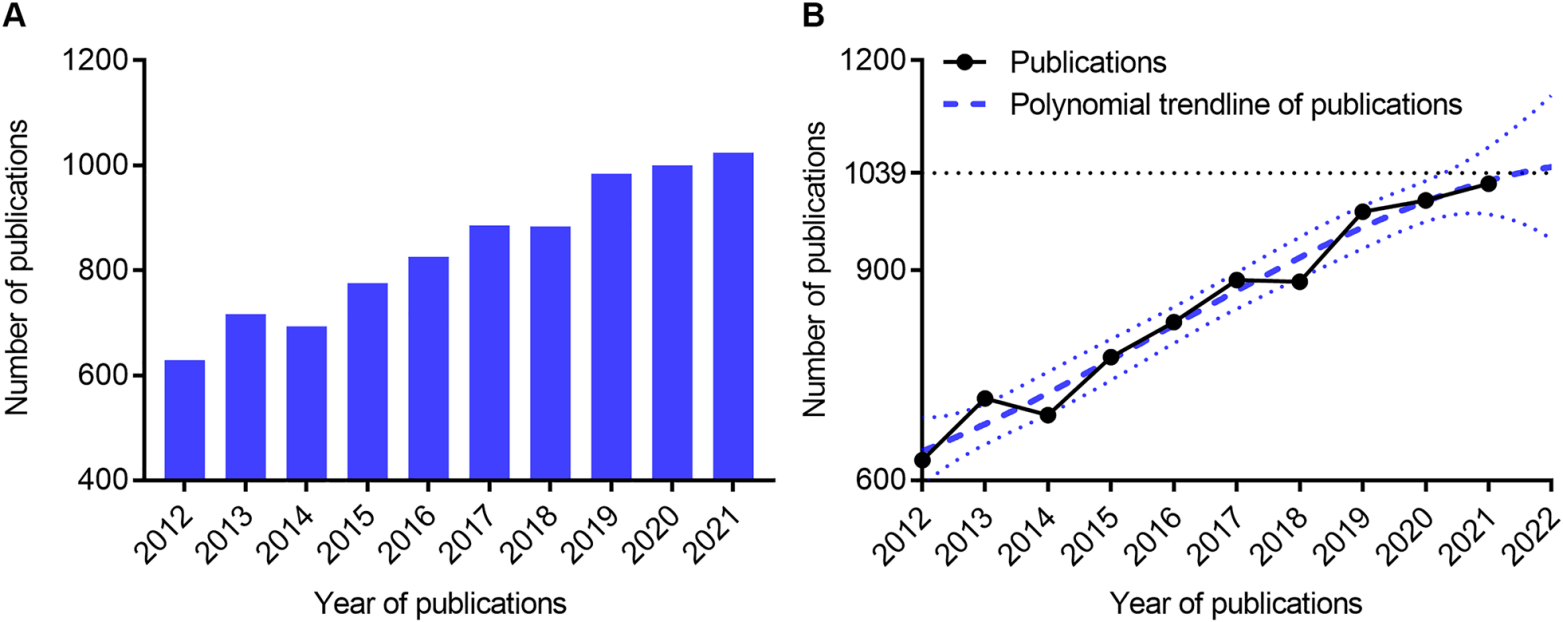
Publication outputs and growth forecast of MI/R injury research. **A** Annual number of publications from 2012 to 2021. **B** Polynomial curve fitting of publication growth. The fitting formula is ${\text{y}}\, = \,846.7\, + \,49.32\,\, \times \,\,\left( {{\text{x}} - 2017} \right) - 0.5795\,\, \times \,\,\left( {{\text{x}} - 2017} \right)^{2} {-}0.3157\,\, \times \,\,\left( {{\text{x}} - 2017} \right)^{3}$

As presented in Fig. 2A, the number of studies on MI/R demonstrated an overall upward trend rising from 629 publications in 2012 to 1024 publications in 2021. According to the polynomial curve fitting of publication growth, the number of publications was estimated to reach 1039 in 2022 (Fig. 2B). The above results demonstrate that MI/R-related studies have received widespread attention from scholars across the world in recent years.

### Distribution of countries and institutions

**Fig. 3:**
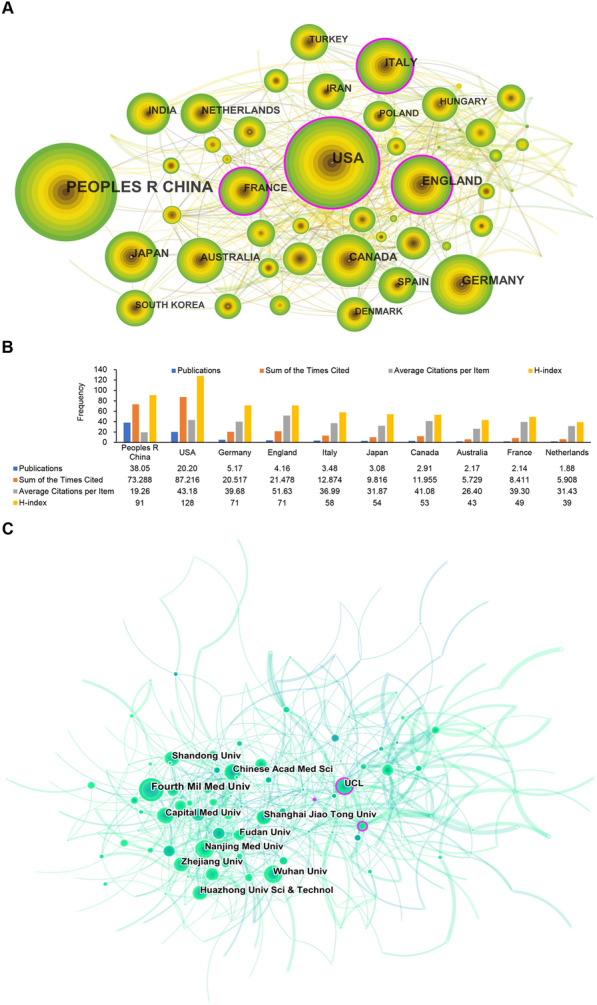
Distribution of countries and institutions involved in MI/R injury research. **A** Network map of countries produced by the CiteSpace tool. **B** Number of publications, total citations, average citations, and H-index of the top 10 countries. **C** Visual network of institutions produced by the CiteSpace tool. The H-index indicates a given country has published at least H publications on MI/R injury from 2012 to 2021 with at least H citations each. Each node represents a country or institution, and the size of the node indicates the publication output of the country or institution. The lines between the nodes represent cooperation between countries or institutions, and the thicker the lines, the closer the cooperation becomes. The color of the node and line signifies different time intervals, and high centrality (> 0.1) is represented by nodes with purple rings

All the studies were distributed among 91 countries and 4847 institutions. As illustrated in Fig. 3A, the largest number of publications originated in China [3805 (45.20%)], followed by the USA [2020 (23.99%)], Germany [517 (6.14%)], England [416 (4.94%)], and Italy [348 (4.13%)]. The top four countries, in terms of centrality, were the USA (0.20), France (0.19), England (0.17), and Italy (0.13), representing close cooperation among themselves. The country with the most average citations was England (51.02). With regard to the H-index, the USA (128), China (91), England (71), Germany (71) and Italy (58) were the top five countries (Fig. 3B).

As depicted in Fig. 3C, Fourth Military Medical University [188 publications (2.23%)], Nanjing Medical University [138 publications (1.64%)], Capital Medical University [131 publications (1.56%)], Wuhan University [131 publications (1.56%)], and Shanghai Jiao Tong University [130 publications (1.54%)] were the largest contributors of MI/R research publications. University College London (UCL) had the highest centrality (0.13) among all the institutions, representing itself as a key hub for promoting cooperation among relevant research institutions. These results indicate that the USA, England, China, Germany, and Italy have exerted a strong scholarly influence in the field of MI/R.

### Distribution of authors

A total of 28,814 authors were involved in MI/R research. As presented in Table 1, the author who published the most publications was Peter Ferdinandy from the Semmelweis University (67 publications), followed by Derek J Hausenloy from University College London (63 publications), Erhe Gao from Temple University (55 publications), Derek M Yellon from University College London (53 publications), and Yang Yang from the Fourth Military Medical University (53 publications). The top 10 contributing authors hailed from institutions in Europe (*n* = 6), China (*n* = 3), and North America (*n* = 1). All the top 10 most published authors had total citation counts of over 1000, and their average number of citations per publication was more than 25. In terms of H-index, the top five authors were Derek J Hausenloy, Derek M Yellon, Gerd Heusch, Rainer Schulz, and Erhe Gao.

**Table 1:** Top 10 authors distributed according to publications on MI/R injury from 2012 to 2021

Rank	Authors	Publications	Total citations	Average citations	H-index^#^	Institution	Country
1	Peter Ferdinandy	67	3452	51.52	27	Department of Pharmacology and Pharmacotherapy, Semmelweis University	Hungary
2	Derek J Hausenloy	63	6390	101.43	41	The Hatter Cardiovascular Institute, University College London	England
3	Erhe Gao	55	2213	40.24	30	Center for Translational Medicine, Temple University	The USA
4	Derek M Yellon	53	5650	106.60	38	The Hatter Cardiovascular Institute, University College London	England
5	Yang Yang	53	2291	43.23	25	Department of Cardiovascular Surgery, Xijing Hospital, The Fourth Military Medical University	China
6	Rainer Schulz	51	3365	65.98	30	Institute for Physiology, Justus-Liebig University Giessen	Germany
7	Zhengyuan Xia	51	1297	25.43	23	Department of Anesthesiology, The University of Hong Kong	China
8	Hans Erik Botker	48	2782	57.96	24	Department of Cardiology, Aarhus University Hospital	Denmark
9	Gerd Heusch	44	5306	120.59	31	Institute of Pathophysiology, University of Essen Medical School	Germany
10	Jian Yang	43	1261	29.33	22	Department of Cardiology, the First College of Clinical Medical Sciences, China Three Gorges University	China

^#^The H-index indicates a given author has published at least H publications on MI/R injury from 2012 to 2021 with at least H citations each

**Fig. 4:**
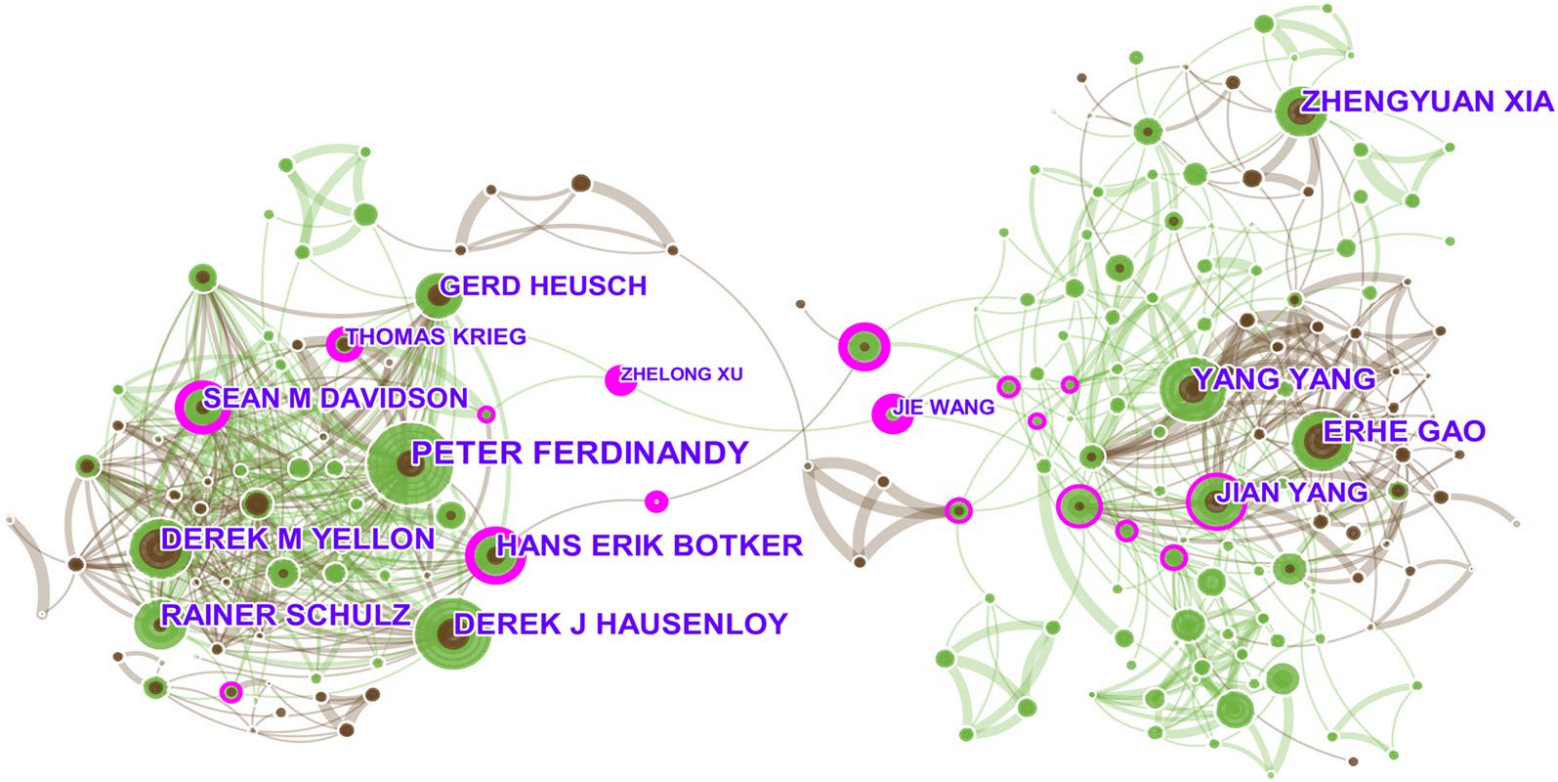
Author collaboration network based on publications related to MI/R injury. The size of the nodes represents the number of papers published by the author, and the connections between the nodes reflect the strength of the collaboration relationship. The nodes with high centrality (> 0.1) are highlighted with purple rings. The brown and green lines represent the first co-occurrence of authors in 2012–2016 and 2017–2021, respectively

The network of author cooperation is presented in Fig. 4. The highest ranked authors, according to centrality, were Jie Wang from Tianjin Medical University (0.42), Zhelong Xu from Tianjin Medical University (0.41), Thomas Krieg (0.29) from University of Cambridge, Sean M Davidson from University College London (0.24), and Hans Erik Botker from University College London (0.22).

### Distribution of journals

The documents were published in 1203 journals, 13 of which had more than 100 publications (Table 2). *PloS One* accounted for the most output, followed by *Oxidative Medicine and Cellular Longevity*, *Molecular Medicine Reports*, *International Journal of Molecular Sciences*, and *Journal of Molecular and Cellular Cardiology*. Among the top 10 journals with the most publications, *Basic Research in Cardiology* had the highest IF (17.165), ranking sixth in terms of number of publications. Most of the productive journals were classified as Q1 or Q2 according to the 2020 Journal Citation Reports. In addition, the number of studies on MI/R injury was normalized to the sum of publications in respective journals. These results showed that the proportion of studies on MI/R injury was highest in *Basic Research in Cardiology* (22.29% of the total publications per journal), followed by *Journal of Molecular and Cellular Cardiology* (7.11% of the total publications per journal), *Cardiovascular Research* (5.81% of the total publications per journal), *American Journal of Physiology-Heart and Circulatory Physiology* (4.04% of the total publications per journal), and *Oxidative Medicine and Cellular Longevity* (2.93% of the total publications per journal).

**Table 2:** Top 10 journals distributed according to publications and citations

Rank	Journal	Publications	IF (JCR 2020)	JCR quartile	Journal	Total citations	Average citations	IF (JCR 2020)	JCR quartile
1	*PloS One*	240 (2.85%)	3.240	Q2	*Circulation*	6052	137.55	29.690	Q1
2	*Oxidative Medicine and Cellular Longevity*	155 (1.84%)	6.543	Q2	*Circulation Research*	5432	64.67	17.367	Q1
3	*Molecular Medicine Reports*	153 (1.82%)	2.952	Q3	*Cardiovascular Research*	5241	46.38	10.787	Q1
4	*International Journal of Molecular Sciences*	149 (1.77%)	5.923	Q1	*American Journal of Physiology-Heart and Circulatory Physiology*	5018	40.47	4.733	Q1
5	*Journal of Molecular and Cellular Cardiology*	146 (1.73%)	5.000	Q2	*Journal of Molecular and Cellular Cardiology*	4540	31.10	5.000	Q2
6	*Basic Research in Cardiology*	138 (1.64%)	17.165	Q1	*Journal of Biological Chemistry*	4155	115.42	5.157	Q2
7	*American Journal of Physiology-Heart and Circulatory Physiology*	124 (1.47%)	4.733	Q1	*PloS One*	4010	16.71	3.240	Q2
8	*European Journal of Pharmacology*	119 (1.41%)	4.432	Q2	*Proceedings of the National Academy of Sciences of the United States of America*	3591	224.44	11.205	Q1
9	*Frontiers in Pharmacology*	116 (1.38%)	5.810	Q1	*Journal of Clinical Investigation*	3561	254.36	14.808	Q1
10	*Cardiovascular Research*	113 (1.34%)	10.787	Q1	*Journal of the American College of Cardiology*	3419	148.65	24.094	Q1

**Fig. 5:**
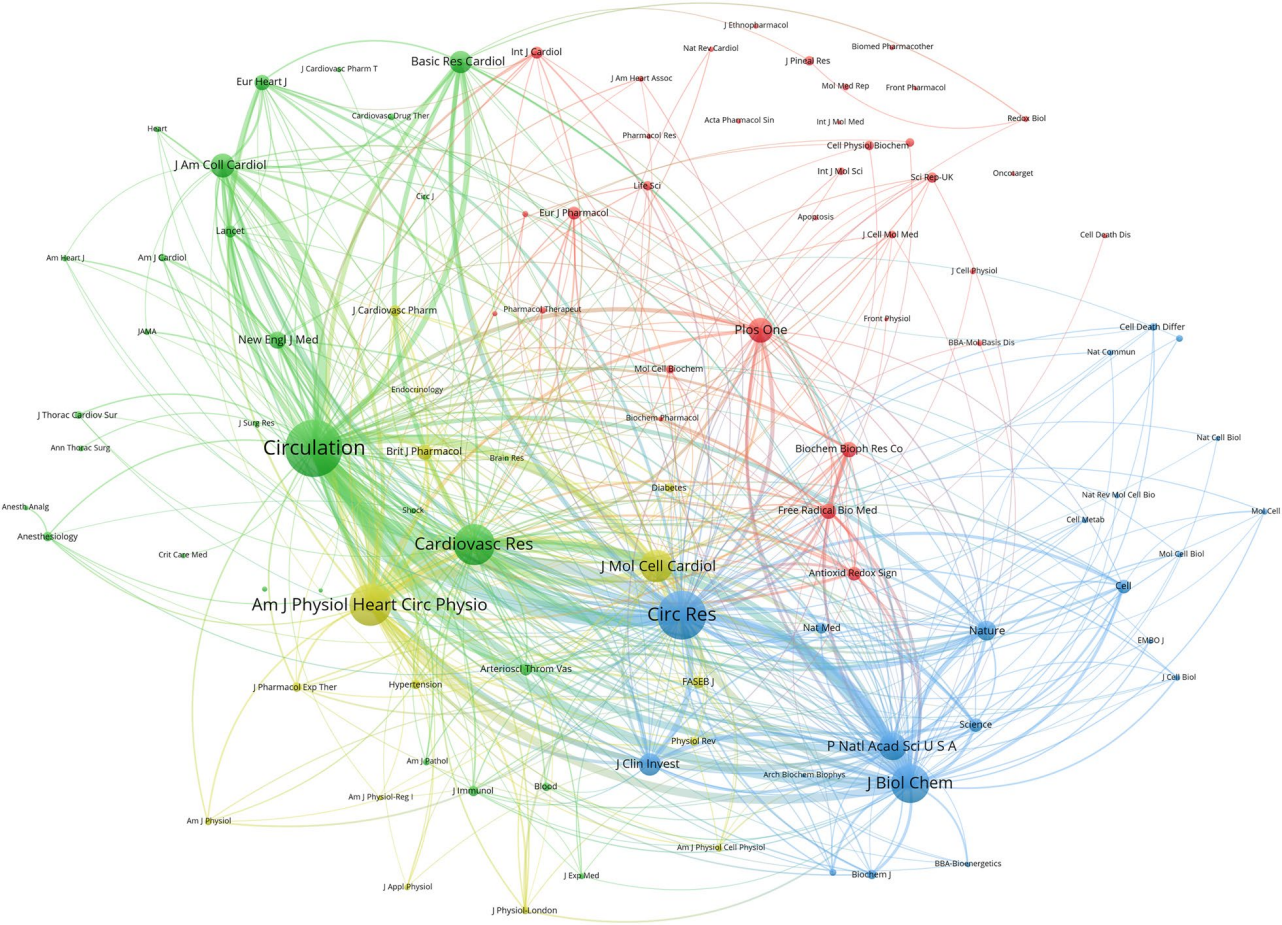
Network map of co-cited journals produced by the VOSviewer tool. The size of the node indicates the co-occurrence frequencies of journals, the link reflects the co-occurrence relationship between journals, and the same color of node represents the respective cluster class

A co-citation relationship between two journals arises when they are both cited in the same publication simultaneously. High co-citation counts imply that the journals produce superior academic material and can, therefore, be considered mainstream. As illustrated in Fig. 5, the top five co-cited journals were *Circulation*, *Circulation Research*, *Cardiovascular Research*, *American Journal of Physiology-Heart and Circulatory Physiology*, and *Journal of Molecular and Cellular Cardiology*. The average IF of the top 10 co-cited journals was 12.608 higher than that of the top journals with the most publications (6.659).

### Analyses of highly cited studies and co-cited references

As presented in Table 3, the top three cited studies were:

1. “Ischaemic Accumulation of Succinate Controls Reperfusion Injury Through Mitochondrial ROS” by Edward T Chouchani in 2014 (1235 citations) [17]. The authors conducted a liquid chromatography/mass spectroscopy-based metabolomics analysis to identify the metabolic pathways responsible for generating mitochondrial reactive oxygen species (ROS) during ischemia/reperfusion injury. This study discovered succinate as a key biochemical basis of mitochondrial ROS production. The authors proposed specific therapeutic targets for MI/R injury through the inhibition of ischemic succinate accumulation and succinate oxidation after subsequent reperfusion.
2. “Myocardial Ischemia–Reperfusion Injury: a Neglected Therapeutic Target” by Derek J Hausenloy in 2013 (1231 citations) [18]. This is a review that discusses the definition and pathophysiological mechanism of MI/R injury. Moreover, the authors summarize studies on therapeutic strategies for reducing MI/R injury, and highlight that preventing MI/R injury has important clinical relevance.
3. “Physiological Implications of Hydrogen Sulfide: a Whiff Exploration That Blossomed” by Rui Wang in 2012 (1226 citations) [19]. This comprehensive review addressed the physiological importance of hydrogen sulfide as an endogenous signaling molecule in related diseases. As for MI/R research, the authors provide insights into the protective effect of hydrogen sulfide against myocardial damage.

**Table 3:** Top 10 high-cited literatures related to MI/R injury from 2012 to 2021

Rank	Total citations	First author	Title	Journal	Year	Column	Page
1	1235	Edward T Chouchani	Ischaemic accumulation of succinate controls reperfusion injury through mitochondrial ROS	*Nature*	2014	515	431–435
2	1231	Derek J Hausenloy	Myocardial ischemia–reperfusion injury: a neglected therapeutic target	*Journal of Clinical Investigation*	2013	123	92–100
3	1226	Rui Wang	Physiological implications of hydrogen sulfide: a whiff exploration that blossomed	*Physiological Reviews*	2012	92	791–896
4	1057	Theodore Kalogeris	Cell biology of ischemia/reperfusion injury	*International Review of Cell and Molecular Biology*	2012	298	229–317
5	981	Y Xie	Ferroptosis: process and function	*Cell Death and Differentiation*	2016	23	369–379
6	754	Sumanth D Prabhu	The biological basis for cardiac repair after myocardial infarction: from inflammation to fibrosis	*Circulation Research*	2016	119	91–112
7	727	Russel J Reiter	Melatonin as an antioxidant: under promises but over delivers	*Journal of Pineal Research*	2016	61	253–278
8	723	Nikolaos G Frangogiannis	The inflammatory response in myocardial injury, repair, and remodelling	*Nature Reviews Cardiology*	2014	11	255–265
9	696	Nikolaos G Frangogiannis	Regulation of the inflammatory response in cardiac repair	*Circulation Research*	2012	110	159–173
10	688	Fatih Arslan	Mesenchymal stem cell-derived exosomes increase ATP levels, decrease oxidative stress and activate PI3K/Akt pathway to enhance myocardial viability and prevent adverse remodeling after myocardial ischemia/reperfusion injury	*Stem Cell Research*	2013	10	301–312

However, it is important to note that the number of citations does not adequately reflect the knowledge base of a particular field, since older articles are typically cited more frequently than newer ones.

**Fig. 6:**
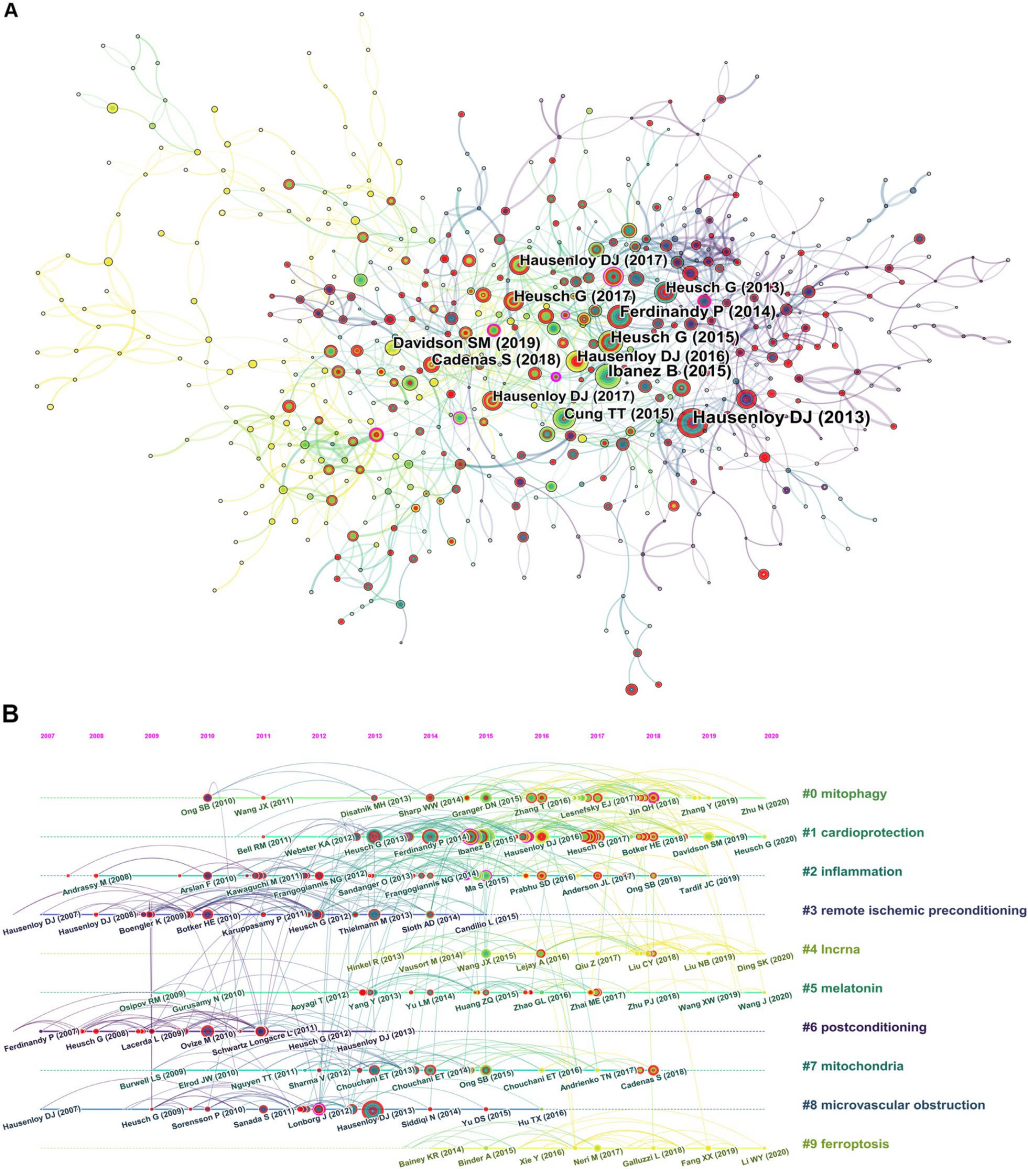
Analysis of co-cited references related to MI/R injury. **A** Co-cited reference network produced by the CiteSpace tool. **B** Timeline view of the top 10 clusters in the co-cited reference network. The size of the nodes reflects the co-citation times of papers. Links are colored based on the date of the first co-citation of the two papers. The color of the node and line represents different years. Nodes with a high centrality (> 0.1) are displayed with purple rings. Nodes with citation bursts are highlighted with red dots or circles. Cluster terms are listed on the right and arranged by the size of the cluster

Reference co-citation analysis identifies highly co-cited references that are frequently cited together by other articles, and is, therefore, usually applied for exploring research foci in a given academic field. A reference co-citation network with 685 nodes and 1857 connections is presented in Fig. 6A. Of the top 10 co-cited references, one is an original article and nine are reviews (Additional file 1: Table S1). “Myocardial Ischemia–Reperfusion Injury: A Neglected Therapeutic Target” by Derek J Hausenloy published in *Journal of Clinical Investigation* was the most frequently co-cited publication [18].

A paper with strong citation burst can be viewed as representing a major milestone in the knowledge base. Figure 6A highlights the papers with citation bursts as red dots or circles. All the top 10 co-cited references had a burst strength of more than 10, except the review published by Sean M Davidson titled “Multitarget Strategies to Reduce Myocardial Ischemia/Reperfusion Injury: JACC Review Topic of the Week” [3]. However, the review published in *Journal of the American College of Cardiology* by Sean M Davidson in 2019 is the latest of the top 10 co-cited references, and therefore, the time lag between publication and citation might have impacted the burst detection.

To further explore characteristics of the temporal evolution of the knowledge base in MI/R research, we performed a cluster analysis using a timeline view tool of CiteSpace (Fig. 6B). The *Q* value of the network of co-cited references was 0.779, indicating that it obtained a good clustering. The average *S* value of the network was 0.933, suggesting high homogeneity of the references in a cluster. Among the top 10 clusters, the clusters which were most recently active included #4 lncrna (long non-coding RNA) and #9 ferroptosis. The clusters #0 mitophagy, #1 cardioprotection, #2 inflammation, #5 melatonin, and #7 mitochondria emerged as active areas in the period 2013–2018, while activity in the five clusters appeared to be declining. The clusters #3 remote ischemic preconditioning, #6 postconditioning, and #8 microvascular obstruction were formed by documents with a mean publication year of 2010, thereby representing older clusters in the MI/R knowledge base.

### Analysis of keywords

**Fig. 7:**
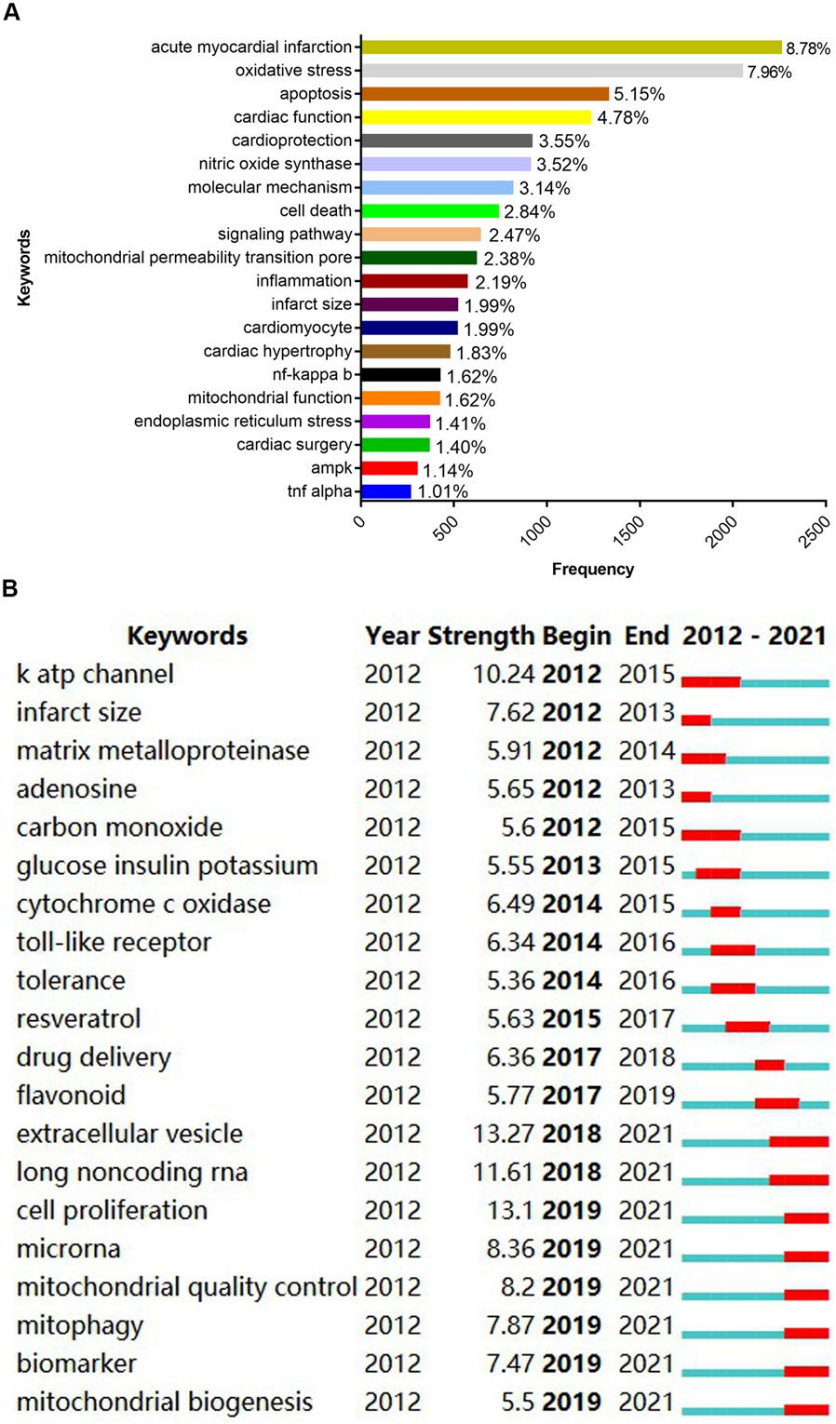
Analysis of keywords in MI/R injury research using the CiteSpace tool. **A** Top 20 keywords with high frequency of co-occurrence. **B** Top 20 keywords with the strongest citation bursts. *Year* represents the time when the keyword first appears. Strength describes the extent of the cited change. *Begin* and *end* indicate the time when each burst starts and ends. The red bar shows the time when the keyword occurs frequently, and the blue bar indicates the time when the keyword occurs rarely

Keywords are the focus of attention in a given scientific paper. A total of 25,662 keywords were extracted by CiteSpace, of which 45 appeared more than 100 times. According to their co-occurrence frequency, the top 20 keywords in MI/R research are listed in Fig. 7A. The high-frequency keywords can be divided into three broad categories: clinical manifestation, molecular mechanism, and cardioprotective strategies for MI/R injury.

To track hotspots and future directions in the field of MI/R research, we further used the burst detection algorithm to analyze the keywords. A keyword with a high burst level indicates that it received greater attention in a specific time period. The top 20 keywords with the strongest appearance bursts are presented in Fig. 7B. Overall, two stages were identified; the first stage lasted from 2012 to 2017, and the second stage spanned from 2018 to 2021. In the first stage, the top keywords were ATP-sensitive potassium (K-ATP) channel, infarct size, matrix metalloproteinase, adenosine, glucose insulin potassium, cytochrome c oxidase, toll-like receptor, tolerance, resveratrol, drug delivery, and flavonoid. The top keywords in the second stage included extracellular vesicles, long noncoding RNA, cell proliferation, microRNA, mitochondrial quality control, mitophagy, biomarker, and mitochondrial biogenesis. These results indicate that the research focus had gradually shifted to acquire an in-depth insight into the pathogenesis underlying MI/R injury.

## Discussion

In the present study, we used bibliometric tools to analyze studies on MI/R injury through the following aspects: (1) the general information was identified and quantified to evaluate individual impact and cooperation information, (2) a co-cited reference analysis was performed to map the knowledge domain, and (3) a keyword analysis was conducted to detect emerging trends. Our findings provide a strong basis for exploring the corpus of knowledge in the MI/R domain.

### General information

One of the indications of the direction of development in a field is change in annual output. According to the WoSCC database, the 8419 papers were published in 1203 academic journals by 28,814 authors from 4847 institutions across 91 countries/regions. The overall trend between 2012 and 2021 was a slow increase in publications. Among the 10 highest output countries, China was the only one from the developing world. The USA was ahead of other countries/regions in H-index, while England had the highest average number of citations per publication. Moreover, the top six authors with the highest number of published articles were from European countries. This implies that the European countries exert a positive academic influence on MI/R research. The most active collaborations were observed among the USA, France, England, and Italy. Nine of the top 10 most prolific institutions were from China, demonstrating its fast progress in MI/R research over the past decade. However, the cooperation among Chinese institutions was infrequent, and UCL in England was the main collaborating center. Good partnerships and a high degree of collaboration among countries and institutions are important for high-quality research output. Therefore, Chinese researchers are encouraged to cultivate partnerships with institutions across the globe as their research output continues to expand, which will improve their academic influence further.

Highlighting the contributions of productive and influential researchers could act as a reference for those scholars and institutions looking for potential collaborators on the basis of research directions [11]. On analyzing author distribution, we found that the top three most influential authors according to the H-index were Derek J Hausenloy (University College London, England), Derek M Yellon (University College London, England), and Gerd Heusch (University of Essen Medical School, Germany). These authors published a large number of papers and were also recognized as the Clarivate Highly Cited Researchers in 2021. The above findings suggest that these three authors have made outstanding contributions, and enjoy a strong academic reputation in the field of MI/R research. Furthermore, the map of authors provides information about potential collaborators who were regarded as influencing authors in Europe and Asia.

In terms of a journal’s publications, IF, and ranking, *Basic Research in Cardiology* is a high-quality journal that exerts considerable influence in the field of MI/R injury. However, mainstream journals in specific fields of study need not have high publication capacity. In fact, we found that some journals with high publications did not receive much attention from the academic community. Quality and influence of a journal can also be determined by its co-citation frequency. The top three journals, in terms of co-citation frequency, were *Circulation*, *Circulation Research*, and *Cardiovascular Research*, with IF values greater than 10. This suggests that these three journals influence research foci, and should thus be considered when tracking progress in MI/R research.

### Knowledge base

The highly co-cited references can be viewed as the knowledge base in a particular field [11]. References with strong citation bursts could also characterize research foundations of a field [11]. Our reference co-citation analysis brought forward some of the influential MI/R studies with highly co-cited counts and strong citation bursts published between 2012 and 2021. In general, the top 10 co-cited references focused on: (1) the pathophysiology of MI/R, including the spatial and temporal evolution of ischemic and reperfusion damage, the cell death modes involved, and consequent coronary microvascular dysfunction, (2) the cardioprotective role of pharmacological and non-pharmacological strategies, such as local ischemic conditioning, remote ischemic conditioning and cyclosporine, and their underlying signal transduction pathways, and (3) potential reasons for failing to translate the extensive and promising basic scientific findings into successful clinical trials. The results offer readers a map for locating those studies that have made large contributions to the field of MI/R research.

Meanwhile, a cluster analysis was conducted to identify the major subfields in the MI/R research, and a timeline view was used to show the developmental process of these subfields. Over the past decade, there has been considerable progress in the understanding of mechanisms of MI/R injury, and development of strategies to protect the myocardium from ischemia and reperfusion damage [2, 3, 8]. By analyzing the top keywords and co-cited references, we found that the mechanistic research before 2018 had mostly focused on molecules involved in cell death, inflammation, and coronary microvascular obstruction. Apoptosis is a cell death mode characterized by DNA fragmentation, but no inflammatory response. During MI/R, both mitochondria and endoplasmic reticulum are crucial organelles that mediate cardiomyocyte apoptosis. It has been suggested that oxidative stress, mitochondrial permeability transition pore, and the K-ATP channel are important factors that contribute to the mitochondria-dependent apoptotic pathway in cardiomyocytes [20]. MI/R can affect the protein-folding capacity of the endoplasmic reticulum, and activate the endoplasmic reticulum stress response [21]. While endoplasmic reticulum stress initially serves adaptive purposes for restoring proteostasis, severe or persistent stress leads to cell apoptosis. Accumulated evidence implicates that the inhibition of excessive endoplasmic reticulum stress could improve cardiac function following MI/R injury [21, 22]. When MI/R induces necrotic cell death, an inflammatory cascade continues damaging the cardiac tissue [23]. The transcription factor nuclear factor-κB (NF-κB) is a key player in the inflammatory signaling. Previous basic research reported that inhibiting the NF-κB pathway minimizes the release of inflammatory factors and reduces infarct size, thereby protecting against MI/R injury [23]. Pyroptosis is a unique form of programmed cell death, that triggers inflammatory response upon infection or sterile insults [24, 25]. During MI/R, a wide range of danger signals, such as oxygen-free radicals, potassium efflux, and mitochondrial stress are produced by dying cells, which activate the inflammasome protein complex leading to pyroptotic cell death and interleukin-1β-driven inflammation [26, 27]. Experimental data shows that inhibition of inflammasome activation could reduce infarct size and cardiac dysfunction following MI/R [28, 29]. Ischemia/reperfusion injury in the coronary circulation manifests as microvascular obstruction and intramyocardial hemorrhage [30, 31]. The coronary microcirculation plays a crucial role in tissue oxygenation and nutrition exchange, and is an important determinant of the infarct size after MI/R [30, 32]. Experimental studies have shown that melatonin treatment protects cardiac microvasculature against MI/R injury by activating the AMPK pathway [33].

As for cardioprotective strategies, experimental studies in the early years from 2007 to 2012 had revealed the essential signal transduction pathways underlying local ischemic postconditioning and remote ischemic preconditioning [34]. For example, oxidative stress after ischemia/reperfusion produces a large number of reactive oxygen and nitrogen species, leading to cardiac cell death and contractile dysfunction. Regulation of redox balance has been demonstrated as an important target of these conditioning strategies against MI/R injury [35–37]. In the intermediate years from 2013 to 2018, MI/R research had focused on translating the cardioprotection of conditioning strategies through animal experiments and early proof-of-concept trials in humans with acute myocardial infarction to clinical practice. In addition, a variety of pharmacological approaches to cardioprotection, such as adenosine and glucose insulin potassium, have progressed to clinical testing for treatment of MI/R damage. Unfortunately, most clinical studies fail to provide evidence of the cardioprotective effects of conditioning strategies and pharmacological approaches [2, 38]. There are several reasons why clinical translation fails, and here we present three major ones [39]:

1. The pathophysiological mechanism of MI/R injury is complex, and understood only partially. Besides causing cardiomyocyte death via multiple mechanisms, MI/R also affects other types of cells, such as platelets, fibroblasts, endothelial cells, smooth muscle cells, and immune cells. Cardioprotective strategies that target a single mechanism might be insufficient at a time when clinical situations usually present a number of uncontrolled variables.
2. The majority of preclinical experiments are conducted on young and healthy animals with a virgin coronary circulation (free of atherosclerosis and endothelial dysfunction). The routinely used animal models do not adequately mimic the clinical scenario of MI/R injury in patients with co-morbidities and co-medications, such as aging, hyperglycemia, hypertension, hyperlipidemia, and the use of P2Y12 antagonists [3].
3. Translating novel cardioprotective interventions into clinical practice is becoming increasingly challenging due to favorable outcomes after optimal reperfusion therapy. It could be possible that the specific population in a clinical trial did not include those patients who would benefit most from cardioprotective interventions.

Although all attempts have failed, better adjunct cardioprotection is still a requirement for decreasing the risk of heart failure after acute myocardial infarction [9]. A clinical translation can be successful and improved through better understanding of the pathophysiology of MI/R injury and more thorough and reliable preclinical evaluation [2, 9]. As seen in the current study, the knowledge base of MI/R from 2019 to 2021 turned to research providing new insights into the molecular events triggered by MI/R, such as lncRNA and ferroptosis. However, few studies were performed on animal models with multiple comorbidities receiving “background drug treatment”. Future studies should be based on robust preclinical data to provide effective translations of cardioprotective strategies from experimental to clinical settings [9].

### Research frontiers

The keywords with strongest citation bursts were analyzed to present those research frontiers that have recently attracted close attention from the academic community [11]. The keywords with strongest citation bursts (until 2021) identified by this study were mitochondrial quality control, non-coding RNAs, cell proliferation, and extracellular vesicles.

Mitochondria are abundantly distributed and plentiful in the heart tissue. Despite the canonical role of mitochondria in regulating cellular respiration and oxidative phosphorylation, recent studies have concluded that mitochondria are crucial in sensing and integrating extracellular and intracellular signals [40]. There is strong evidence that mitochondrial dysfunction is a decisive signal in various modes of cardiomyocyte death during MI/R injury [41]. Pharmacological agents targeting mitochondrial function, such as cyclosporine A, TRO-40303, and MTP-131 have been shown to attenuate cardiac insults and improve contractile function in preclinical animal models. However, the mitoprotective agents as adjunct to reperfusion have not yet translated into clinically beneficial treatments for patients with acute myocardial infarction [42]. The partial reason for this failure could be due to insufficient pathophysiological knowledge of mitochondria. Evidence from recent studies indicates that mitochondria have a quality control system that maintains and restores their structure and function by regulating mitochondrial biogenesis, networks (fission or fusion), and degradation (mitophagy). If the severity of a stimuli overcomes the protective capacity of the quality control system, mitochondria-dependent cell death would be activated to remove the damaged cells. Abnormal mitochondrial quality control is closely related to mitochondrial dysfunction and contributes to MI/R damage [43]. Experimental data suggest that mitochondrial fission and fusion proteins, which play vital roles in the process of mitochondrial quality control, have emerged as novel targets for cardioprotection [43]. Further studies using clinically relevant large animal models of MI/R injury are needed to test whether targeting mitochondrial fission and fusion proteins such as dynamin-related protein 1 (DRP1) and optic atrophy 1 (OPA1) could preserve mitochondrial function and have therapeutic potential to reduce infarct size. These may be the new foci in the field of MI/R research.

In ongoing research on the regulatory mechanisms of MI/R injury, the focus has gradually shifted from coding RNAs to non-coding RNAs (ncRNAs), particularly microRNAs, lncRNAs, and circular RNAs [44]. microRNAs primarily interact with the 3'-untranslated region of the target mRNA, thus preventing protein translation through RNA-interference [45]. lncRNAs can alter gene expression networks in the nucleus and modulate mRNA stability, translation, and protein function in the cytoplasm by directly interacting with RNA, DNA, or protein [46, 47]. The regulatory actions of circular RNAs include host gene regulation, scaffold, and molecular sponge function [48]. ncRNAs can influence the cellular function of all types of cardiac cell, and are critical regulators during MI/R injury [44]. ncRNAs have also been identified as important diagnostic and prognostic biomarkers in cardiac diseases, including MI/R injury. Beyond this, ncRNAs are rapidly developing as targets (e.g., microRNAs, lncRNAs) and tools (e.g., small interfering RNAs) in novel therapeutic strategies. Considering that about 95% of the human genome produces a vast number of ncRNAs with versatile modes of action in the cell, current understanding of the regulation of MI/R injury by ncRNAs is still rather limited [44]. Further studies are required to develop a deeper understanding of the mechanisms and functions of ncRNAs.

Loss of cardiomyocytes is the major contributor to the pathogenesis of MI/R injury [49]. It is well-known that mammalian cardiomyocyte proliferation is active during development and decreases in the early neonatal stage. Recent studies on cardiomyocyte proliferation have overturned traditional beliefs regarding adult cardiomyocytes permanently leaving the cell cycle [49, 50]. By stimulating the endogenous capacity of cardiomyocyte proliferation, the adult heart can achieve myocardium regeneration and meaningful functional recovery after MI/R injury [8, 50, 51]. However, these findings mainly resulted from in vitro experiments or non-mammalian and rodent in vivo studies. Research involving higher animal models or human trials relied on surrogate measures rather than true cardiomyogenesis assessment [51]. Extensive experiments with large mammals and rigorous assessment of cardiomyogenesis are required to advance the notion of cardiac regeneration toward clinical application [51].

Extracellular vesicles are nanosized vesicles with a double-layer lipid membrane. There is increasing evidence that reveals the vital role of extracellular vesicles as cell-to-cell communication vehicles in the development of MI/R injury [52]. Exosomes, a subtype of extracellular vesicles, are released by various types of cells, and are loaded with non-random subsets of bioactive molecules (proteins, lipids, ncRNAs, and mRNAs) that exist in their source cells. Stem cell-derived exosomes have been shown to confer cardioprotective effects, activate regenerative signals, and participate in cardiac repair after MI/R injury [52]. The biological role and diagnostic and therapeutic potential of extracellular vesicles are attracting increasing attention from researchers in the MI/R field.

### Strengths and limitations

This study found that the MI/R knowledge base has evolved rapidly over the past 10 years. Early MI/R research was initially focused on pharmacological and non-pharmacological approaches to inducing cardioprotection, and on their signal transduction pathways. However, research has gradually shifted focus to insightful molecular mechanisms triggered by MI/R. The findings of this study would act as a reference for researchers looking for research directions, and lay the foundation for further research on MI/R injury. In addition, our results reveal the most productive authors and institutions, which could help researchers decide on collaborations. Journals publishing high-impact studies should be followed by researchers to track the progress of MI/R research.

However, the present study does have several limitations. First, studies were not retrieved from databases other than the WoSCC database (for instance, Google Scholar or PubMed). Second, only English articles and reviews were included, which could have resulted in a language bias. Third, different H-index values are possible for the same author, because the H-index method is based on the number of publications and citations in a given time period.

## Conclusions

This study used a bibliometric analysis to provide researchers with a panoptic and holistic perspective on research in the field of MI/R injury. The number of publications on MI/R research has increased over the past decade. Research foci in the MI/R field are the pathophysiology of MI/R injury and development of strategies to protect the myocardium from ischemia and reperfusion damage. Mechanistic research before 2018 had mainly focused on the molecules or pathways involved in cell death, inflammation, and coronary microvascular obstruction, such as mitochondrial permeability transition pore, endoplasmic reticulum stress, oxidative stress, NF-κB, or AMPK pathways. The latest hotspots are more in-depth insights into the molecular mechanisms underlying MI/R injury, such as mitochondrial quality control, ncRNAs, cell proliferation, and extracellular vesicles. With regard to cardioprotective interventions proven to be beneficial in preclinical animal studies, it has been challenging to translate them into clinically beneficial treatments for patients with acute myocardial infarction. Conducting rigorous and systematic in vivo preclinical assessment, such as adopting an animal model with the coexistence of multiple confounding factors, would increase the likelihood of translating novel cardioprotective interventions into clinical practice for patients’ benefit.

## Supplementary Information

**Additional file 1: Table S1.** Top 10 co-cited references for MI/R injury research. **Fig. S1.** Process of searching, retrieving, and selecting potentially relevant studies.

## Data Availability

All data analyzed during this study are included in this manuscript.

## Abbreviations

MI/R: Myocardial ischemia/reperfusion
WoSCC: Web of science core collection
IF: Impact factor
H-index: Hirsch index
ROS: Reactive oxygen species
lncRNA: Long non-coding RNA
NF-κB: Transcription factor nuclear factor-κB
ncRNAs: Non-coding RNAs
