# Supplementary material for: Insights into research on myocardial ischemia/reperfusion injury from 2012 to 2021: a bibliometric analysis
Source: Eur J Med Res. 2023 Jan 9;28:17. doi: 10.1186/s40001-022-00967-7 (PMC9827672; doi:10.1186/s40001-022-00967-7)
Supplement: Supplementary file 1 — Additional file 1: Table S1. Top 10 co-cited references for MI/R injury research. Fig. S1. Process of searching, retrieving, and selecting potentially relevant studies. [file 40001_2022_967_MOESM1_ESM.pdf]

Table S1 Top 10 co-cited references for MI/R injury research.

Rank	Co-cited Frequency	First Author	Title	Journal	Year	Column	Page
1	325	Derek J Hausenloy	Myocardial ischemia-reperfusion injury: a neglected therapeutic target	*Journal of Clinical Investigation*	2013	123	92-100
2	221	Borja Ibanez	Evolving therapies for myocardial ischemia/reperfusion injury	*Journal of the American College of Cardiology*	2015	65	1454-1471
3	182	Gerd Heusch	Molecular basis of cardioprotection: signal transduction in ischemic pre-, post-, and remote conditioning	*Circulation Research*	2015	116	674-699
4	177	Peter Ferdinandy	Interaction of risk factors, comorbidities, and comedications with ischemia/reperfusion injury and cardioprotection by preconditioning, postconditioning, and remote conditioning	*Pharmacological reviews*	2014	66	1142-1174
5	154	Sean M Davidson	Multitarget strategies to reduce myocardial ischemia/reperfusion injury: JACC review topic of the week	*Journal of the American College of Cardiology*	2019	73	89-99
6	142	Gerd Heusch	The pathophysiology of acute myocardial infarction and strategies of protection beyond reperfusion: a continual challenge	*European Heart Journal*	2017	38	774-784
7	141	Susana Cadenas	ROS and redox signaling in myocardial ischemia-reperfusion injury and cardioprotection	*Free Radical Biology & Medicine*	2018	117	76-89
8	136	Derek J Hausenloy	Ischaemic conditioning and reperfusion injury	*Nature Reviews Cardiology*	2016	13	193-209
9	132	Gerd Heusch	Cardioprotection: chances and challenges of its translation to the clinic	*Lancet*	2013	381	166-175
10	126	Thien-Tri Cung	Cyclosporine before PCI in patients with acute myocardial infarction	*The New England Journal of Medicine*	2015	373	1021-1031

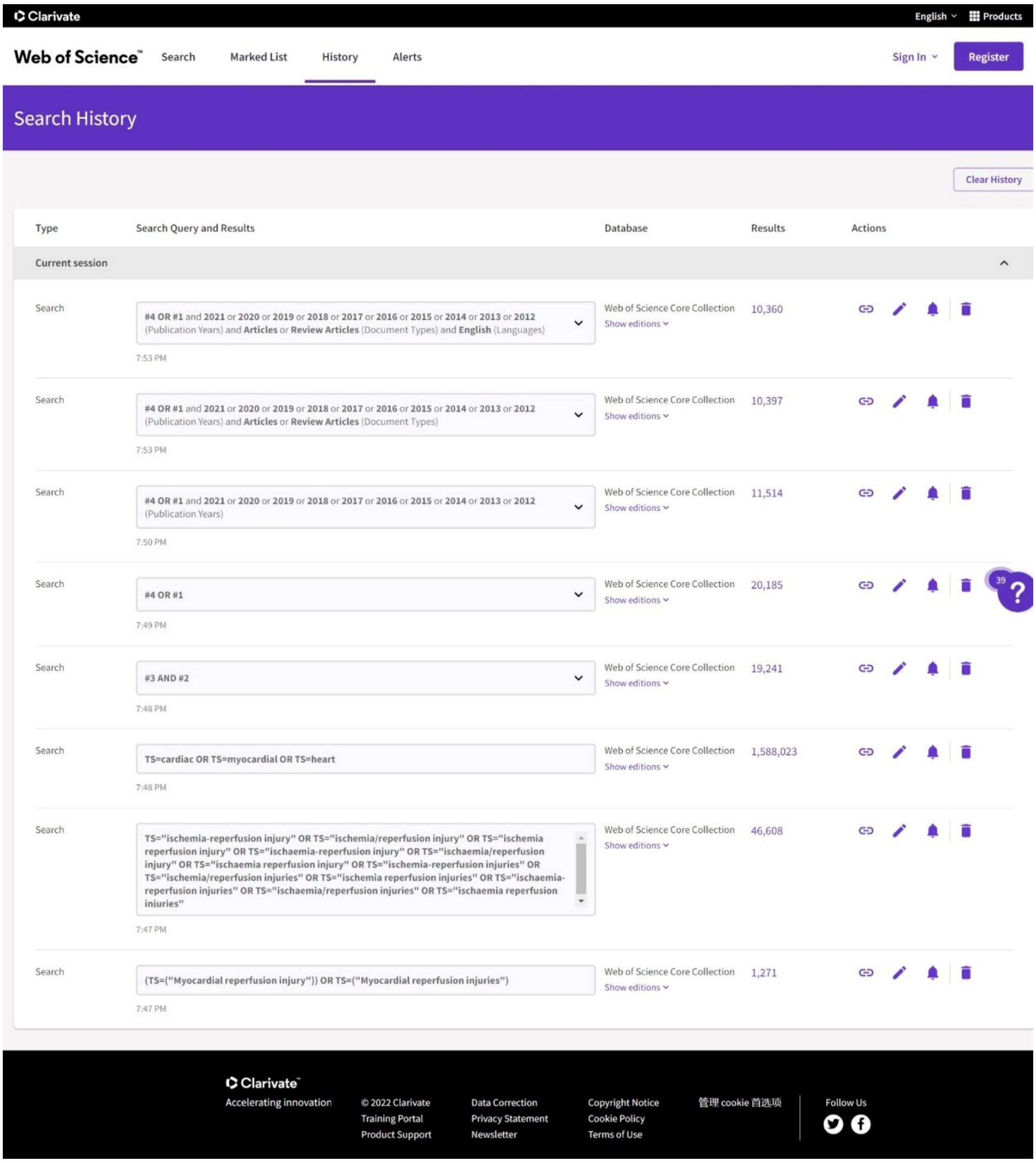

Figure S1 Process of searching, retrieving, and selecting potentially relevant studies.
